# Infection as a potential cofactor in the genetic-epigenetic pathophysiology of endometriosis: a systematic review

**Published:** 2020-01-24

**Authors:** PR Koninckx, A Ussia, M Tahlak, L Adamyan, A Wattiez, DC Martin, V Gomel

**Affiliations:** Latifa Hospital, Dubai, United Arab Emirates;; KU, Leuven, Belgium;; Gruppo Italo-Belga, Villa Del Rosario, Rome, Italy;; University of Oxford-Hon Consultant, Oxford,UK; Università Cattolica, Roma Italy;; Department of Operative Gynecology, V. I. Kulakov Research Centre for Obstetrics, Gynecology, and Perinatology, Russian Federation; Department of Reproductive Medicine and Surgery, Moscow State University of Medicine and Dentistry, Moscow, Russia;; Department of Obstetrics and Gynaecology, University of Strasbourg, France;; School of Medicine, University of Tennessee Health Science Centre, Memphis Tennessee, USA;; Virginia Commonwealth University, Richmond, Virginia, USA;; Department of Obstetrics and Gynecology, University of British Columbia and Women’s Hospital, Vancouver, BC, Canada.

**Keywords:** Endometriosis, pathophysiology, infection, prevention of endometriosis, adolescent endometriosis

## Abstract

**Background:**

The genetic-epigenetic theory postulates that endometriosis is triggered by a cumulative set of genetic-epigenetic (GE) incidents. Pelvic and upper genital tract infection might induce GE incidents and thus play a role in the pathogenesis of endometriosis. Thus, this article aims to review the association of endometriosis with upper genital tract and pelvic infections.

**Methods:**

Pubmed, Scopus and Google Scholar were searched for ‘endometriosis AND (infection OR PID OR bacteria OR viruses OR microbiome OR microbiota)’, for ‘reproductive microbiome’ and for ‘reproductive microbiome AND endometriosis’, respectively. All 384 articles, the first 120 ‘best match’ articles in PubMed for ‘reproductive microbiome’ and the first 160 hits in Google Scholar for ‘reproductive microbiome AND endomytriosis’ were hand searched for data describing an association between endometriosis and bacterial, viral or other infections. All 31 articles found were included in this manuscript.

**Results:**

Women with endometriosis have a significantly increased risk of lower genital tract infection, chronic endometritis, severe PID and surgical site infections after hysterectomy. They have more colony forming units of Gardnerella, Streptococcus, Enterococci and Escherichia coli in the endometrium. In the cervix Atopobium is absent, but Gardnerella, Streptococcus, Escherichia, Shigella, and Ureoplasma are increased. They have higher concentrations of Escherichia Coli and higher concentrations of bacterial endotoxins in menstrual blood. A Shigella/Escherichia dominant stool microbiome is more frequent. The peritoneal fluid of women with endometriosis contains higher concentrations of bacterial endotoxins and an increased incidence of mollicutes and of HPV viruses. Endometriosis lesions have a specific bacterial colonisation with more frequently mollicutes (54%) and both high and medium-risk HPV infections (11%). They contain DNA with 96% homology with Shigella. In mice transplanted endometrium changes the gut microbiome while the gut microbiome influences the growth of these endometriosis lesions.

**Conclusions:**

Endometriosis is associated with more upper genital tract and peritoneal infections. These infections might be co-factors causing GE incidents and influencing endometriosis growth.

## Introduction

Endometriosis is defined as ‘endometrium like cells outside the uterus’. Clinically endometriosis is a hereditary and heterogeneous disease with a variable presentation. It occurs in women without endometrium (Kawano et al., 2014) and in men taking estrogens (Rei et al., 2018) and even in those not taking estrogens (Giannarini et al., 2006; Jabr and Mani, 2014). Endometriosis is associated with pain, infertility, adenomyosis and altered pregnancy outcomes. Women with endometriosis have many biochemical changes in the endometrium, in plasma and in peritoneal fluid. The endometriosis lesions are clonal in origin and they have numerous and variable biochemical alterations such as aromatase activity and progesterone resistance. These observations can be explained by the genetic- epigenetic theory (Koninckx et al., 2019). The set of genetic and epigenetic changes inherited at birth (Simpson et al., 1980; Coxhead and Thomas, 1993; Moen, 1994; Hadfield et al., 1997; Kennedy, 1998; Kennedy et al., 1998; Treloar et al., 1999; Moen and Magnus, 1993) could explain the predisposition, the changes in the endometrium and in plasma (Bulun et al., 2015), the changes in immunology (Herington et al., 2011; PaulDmowski and Braun, 2004; Riccio et al., 2018; Zhang et al., 2018), the decreased defence mechanism against oxidative stress (Asghari et al., 2018) and the associated pregnancy disorders (Koninckx et al., 2018). When in some cells additional genetic or epigenetic incidents reach a certain threshold (Koninckx et al., 2019) the cells start to develop as endometriosis lesions. Further development of these lesions will vary with the specific set of genetic and epigenetic changes, and the environment of the peritoneal cavity or the ovary. This environment is different from the uterine environment by a different immunology, endocrinology, growth factors and cytokines. In addition, outside the uterus there is no junctional zone. Epigenetic and genetic changes can be caused by random errors during cell cleavage and by factors as radiation (Gordts et al., 2017), pollution with dioxins (Bruner-Tran and Osteen, 2010; Guo et al., 2009; Rier and Foster, 2002; Sofo et al., 2015), and oxidative stress (Augoulea et al., 2012; Gupta et al., 2006; Ito et al., 2017; Scutiero et al., 2017). Especially the oxidative stress of blood may be important in the uterine cavity, in the peritoneal cavity after retrograde menstruation (Donnez et al., 2016) and in the endometriosis lesions (Metzger et al., 1988). Also infection (Bierne et al., 2012; Ewald and Ewald, 2012) and viruses (Clarke and Clements, 1991; Perales et al., 2011; Akhter et al., 2014; Milavetz and Balakrishnan, 2015; Zhu et al., 2016) can induce genetic and epigenetic changes. Infection moreover increases oxidative stress and changes immune responses (Campos et al., 2018; Khan et al., 2018), and was suggested to be a cause of endometriosis (Khan et al., 2018).

The important roles of the microbiome of the gut and of the uterus and upper genital tract were only recently realised. The peritoneal cavity and the uterus are not sterile environments but contain specific microbial communities (Chen et al., 2017). The intra-uterine microbiome (Baker et al., 2018) affects embryo implantation as evidenced during IVF (de Ziegler et al., 2016; Moreno et al., 2016). Diet and lifestyle affect the microbiome of the gut (Conlon and Bird, 2014). Gut microbiota supply essential nutrients, synthetize vitamins, and play a role in angiogenesis and epithelial repair. Changes of the gut microbiota contribute to the development and progression of diseases, such as inflammatory bowel diseases, arthritis, psoriasis and cancer. Gut microbiota could influence the development of endometriosis through modulation of the immune responses and of pelvic inflammation (Laschke and Menger, 2016), by metabolising oestrogens (Baker, et al., 2017) and by affecting circulating oestrogen concentrations (Flores, et al., 2012).

The effect of sperm cells, which can attach and transport microbes and chlamydia on their tails (Wolner-Hanssen and Mardh, 1984; Confino et al., 1987; Friberg et al., 1987), on the upper genital tract and pelvic microbiome is poorly investigated. This mechanism of sperm transport of infectious agents was used to explain that the risk of non-gonococcal pelvic inflammatory disease (PID) (Toth, 1987) decreases when cervical mucus was less permeable to spermatozoa during the intake of combined oral contraceptives (Rosenberg, 1991). However, over the last decade this protective effect of combined oral contraceptives has become less clear. Changes in sexual behaviour as suggested by the increase in chlamydia infections (Datta et al., 2018) might affect the incidence of PID. Others suggested that the decrease in PID in women using oral contraceptives might result from fewer diagnoses because of less severe PID symptoms (Barrett and Taylor, 2005; Mitchell and Prabhu, 2013). Also the association of PID and ovarian cancer (Shen et al., 2016; Rasmussen et al., 2017; Zhou et al., 2017; Stewart et al., 2018; Trabert et al., 2018), and the decreased risk of ovarian cancer following tubal ligation (Cibula et al., 2011; Ness et al., 2011; Rice et al., 2012; Sieh et al., 2013; Ylikorkala, 2001) and following oral contraceptive use (Group, 2005; Ness et al., 2000) could be related PID. The unclear association of endometriosis and ovarian cancer (Guo, 2015; Ruderman and Pavone, 2017; Dawson et al., 2018; Muangtan et al., 2018) thus could have infection in common.

Considering the (epi)genetic theory of endometriosis (Koninckx, et al., 2019) and the observations that infections (Bierne et al., 2012) and viruses (Clarke and Clements, 1991; Perales et al., 2011; Akhter et al., 2014, Zhu et al., 2016), especially retroviruses (Kassiotis, 2014) are mutagenic with epigenetic effects (Milavetz and Balakrishnan, 2015) the fast growing literature that links endometriosis with upper genital tract and pelvic infections was reviewed.

## Materials and Methods

We reviewed all published data on the association of human endometriosis and bacterial or viral infections till October 2019. Pubmed was searched for ‘endometriosis [Title] AND (infection OR PID OR bacteria OR viruses OR microbiome OR microbiota OR ‘pelvic inflammatory disease’ OR microbial). The title and if suggestive the pdf of all 384 articles found were hand searched by one author (P.R. Koninckx) for a link between endometriosis and infection. Pubmed was searched for ‘reproductive microbiome’ and the 120 best matched articles out of 1899 were hand searched. All articles of interest were added to a dedicated group in a personal endnote database. Google scholar was searched for ‘reproductive microbiome AND endometriosis’. The first 160 hits were searched for a relationship with endometriosis generating 8 additional articles of interest. A series of very recent non-peer reviewed articles on endometriosis treatment by antibiotics and food intake were not incorporated in this review.

The role of spermatozoa in PID was searched by (sperm OR spermatozoa) AND (PID OR pelvic infection) generating 176 hits and by (sperm OR spermatozoa) AND (flat capillaries OR cervical mucus) generating 1560 hits. Also, these articles were hand searched for evidence of the transport of infectious agents by spermatozoa.

All 31 articles describing a link between endometriosis and infection and or microbiota were incorporated in this manuscript. Since selection was done without eligibility criteria, and since all articles were included, a PRISMA flow chart was not included (Moher et al., 2009).

For clarity, throughout our manuscript we refer to microbiota as the group of microorganisms present in a defined environment, and microbiome refers to the entire habitat, thus including microorganisms, their genomes and the environmental conditions.

## Results

### 

Already in 1960 endometriosis was found in 18 to 50% of women with chronic salpingitis versus a small percent in women with acute infections (Jacobson, 1960).

### Women with endometriosis have more upper genital tract infections

Indeed, a higher incidence of chronic endometritis (Cicinelli et al., 2017; Takebayashi et al., 2014), more severe pelvic inflammatory disease (Elizur et al., 2014), a higher risk of surgical site infection after hysterectomy (Chen et al., 2018) and a higher vaginal pH and more colony forming units of Gardnerella, a-Streptococcus, Enterococci and Escherichia coli in the endometrium (Khan et al., 2014). Women with cystic ovarian and deep endometriosis have in their cervix no Atopobium but an increase in Gardnerella, Streptococcus, Escherichia, Shigella, and Ureoplasma, and more frequently a Shigella/ Escherichia dominant stool microbiome (Ata et al., 2019). Women with endometriosis, especially with red lesions have a higher concentration of Escherichia Coli in menstrual blood and higher concentrations of bacterial endotoxins in menstrual blood and in peritoneal fluid (Khan et al., 2010; Khan et al., 2018). Women with endometriosis have a specific microbial colonisation in the uterus and in the peritoneal fluid (Chen et al., 2017) and in the uterus and fluid of cystic ovarian endometriosis (Khan et al., 2016). They have more frequently Enterobacteriaceae and Streptococcus in their cervical mucus (Akiyama et al., 2019). The significantly altered cervical microbiome in endometriosis stage III becomes normal for a short period after surgery (Cregger et al., 2017).

A history of PID is associated with a 3-fold increased risk of developing endometriosis within 10 years in comparison with control women (Tai et al., 2018). Chronic endometritis is associated with an altered uterine contractility which might increase retrograde menstruation (Pinto et al., 2015). Lower genital tract infection is an independent risk factor for developing endometriosis with and odds ratio of 2 in comparison with a control group (Lin et al., 2016).

### Women with endometriosis have more mollicutes in peritoneal fluid

Mollicutes (mycoplasma hominis, mycoplasma genitalium, ureaplasma urealyticum, and ureaplasma parvum) were found in the peritoneal fluid of women with and without endometriosis in 54% and 33% respectively (Campos et al., 2018). Although not statistically significant when compared individually, the prevalences of mycoplasma hominis, mycoplasma genitalium, ureaplasma urealyticum were systematically higher in peritoneal fluid of women with endometriosis than in women without endometriosis. In peritoneal fluid of women with endometriosis, M. genitalium, M. hominis and U. urealyticum were found whereas in the control group only M. genitalium and M. hominis were detected. In the peritoneal biopsies of women with endometriosis M. genitalium and M. hominis were found whereas in the control group only M. genitalium was detected. Both peritoneal fluid and peritoneal biopsies showed a significant higher diversity in microorganisms in women with endometriosis than in the control group (Campos et al., 2018).

### Endometriosis lesions contain frequently HPVA viruses

Although an initial report did not find increased viral DNA in endometriosis (Vestergaard et al., 2010), more recently high and medium risk human papilloma viruses (HPV), but not herpes virus or chlamydia were found in 11% of the endometriosis lesions and in 27% of other pelvic tissues (Oppelt et al., 2010). High-risk and medium-risk HPV were detected in 26% and 10% of ovaries with and without a cystic ovarian endometriosis (Heidarpour et al., 2017). In a prospective case control study, endometriosis was specifically associated with upper genital tract high risk HPV infection but not with other sexually transmitted diseases (Rocha et al., 2019).

Although the significance remains unclear, endometriosis lesions contain DNA with 96% homology with Shigella DNA (Kodati et al., 2008).

### In mice, induced endometriosis interacts with the gut microbiome and vice-versa

In mice, induction of endometriosis altered the gut microbiota with an increased Firmicutes/ Bacteroidetes ratio (Yuan et al., 2018). In a mouse endometriosis model, the treatment with broad spectrum antibiotics or by metronidazole changed the intestinal microbiome and the growth of ‘endometriosis’ lesions was significantly reduced (Chadchan et al., 2019) unless gut microbiota were restored.

## Discussion

### The specific microbiome of the upper genital tract and of the peritoneal cavity

The peritoneal cavity, the upper genital tract, the endometrial cavity and the cervix have a specific microbiome which seems to be a continuum which is determined by the vaginal microbiome like ascending infections together with the specific local immunity. The microbiome of the peritoneal cavity is in addition influenced by the gut microbiome by transmural migration.

The role of the uterine microbiome in fertility and implantation (Moreno et al., 2016; de Ziegler et al., 2016; Baker et al., 2018; Liu et al., 2019) is beyond the scope of this article. The role of the microbiome of the peritoneal cavity and of the upper genital tract is unclear. This microbiome is considered a primary defence mechanism against infection by pathogens (Huang et al., 2014). The peritoneal microbiome plays a role in the innate immunity through bacterial endotoxins or lipopolysaccharides and the Toll-like receptor 4 (Khan et al., 2018).

### The association of endometriosis with upper genital tract and peritoneal cavity infections

The association of endometriosis and infection started with Sampson who described histological similarities between endometriosis and infectious lesions (Sampson, 1927). Subtle endometriosis lesions have often a mainly inflammatory histology (Cabana et al., 2010; Marsh and Laufer, 2005). Ascending infections are consistent with the observation that endometriosis occurs predominantly in the pelvis, and were recently suggested to cause endometriosis by cell trauma (Canis et al., 2017). The apparent increase in severity of deep endometriosis in the western word (Koninckx et al., 2016), could be linked to the changed sexuality and the associated risk of infection. In addition our life style and increased hygiene (Cypers et al., 2014) were suggested to have induced changes in gut microbiome and auto-immunity and in enterococcal bacteriophages facilitating the transmural migration of gut bacteria (Duerkop et al., 2014).

The cumulative evidence that endometriosis is associated with more vaginal and upper genital tract infection and more PID (Elizur et al., 2014) is strong. Not a single report found an association in the opposite direction. The association of endometriosis with infections adds to the many endometriosis associated events (Koninckx et al., 2019). However, the interpretation of associated events should be done carefully, since for many associated events it is unclear which cause endometriosis, which are a consequence and which are the consequence of a common constitution.

Infections could be the consequence of any of the many endometriosis associated events such as infertility, pain, low grade pelvic inflammation (Halme, 1991; Samimi et al., 2019), more abundant menstruation, menorrhagia, alterations in immunology, changes in the endometrium and pregnancy disorders (Koninckx et al., 2019). In addition, medical therapy with e.g. progestagens will affect the vaginal epithelium and flora, the endometrium and endometrial microbiota and probably the peritoneal fluid.

### Infections could be a co factor causing endometriosis

Bacteria (Bierne et al., 2012, Ewald and Ewald, 2012), viruses (Perales et al., 2011; Clarke and Clements, 1991; Akhter et al., 2014; Milavetz and Balakrishnan, 2015; Zhu et al., 2016) and other micro-organisms can cause genetic and epigenetic incidents in endometrial cells and in cells of the peritoneal cavity. Although not yet identified in the peritoneal cavity or in endometriosis lesions, it cannot be excluded that occasionally other viruses as retroviruses with a strong oncologic potential might find their way to the peritoneal cavity (Kassiotis, 2014). Besides a direct genetic or epigenetic effect, these infectious agents and the increased endotoxins increase the cellular and immunological stress and add to the oxidative stress of retrograde menstruation and of bleedings in the lesions (Kobayashi et al., 2014). Since tubal sterilisation decreases the risk of ovarian cancer, it is tempting to speculate that these infections could also cause tubal epithelial damage and ovarian cancer. Infection thus could be the common denominator to explain the unclear association of endometriosis and ovarian cancer.

Infections could contribute to the growth of endometriosis lesions through their effect on immunity, angiogenic and growth factors in the peritoneal cavity (Koninckx et al., 2016). Mycoplasma genitalium down-regulates genes associated with the inflammatory response (Campos et al., 2018). This together with the secretion of glycodelins (placental protein, PP14) by endometriotic cells (Okamoto et al., 1991) and the subsequent decrease in Natural Killer cell activity (Oosterlynck et al., 1991; Kanzaki et al., 1992) plays a role in the immuno-tolerance towards endometriosis. The stimulation of innate immunity by endotoxins plays a role in the low- grade inflammation of the peritoneal cavity in women with endometriosis (Halme, 1991; Khan et al., 2018; Samimi et al., 2019), which thus could be a co-factor in the growth of endometriosis lesions (Khan et al., 2018).

### Endometriosis and intestinal microbiome

The role of the gut microbiota in endometriosis is still unclear. Transmural migration contributes to the peritoneal microbiome, and the finding of Shigella- like DNA in endometriosis lesions suggest a bowel origin (Kodati et al., 2008). With all limitations of the mouse model of endometriosis it is clear that gut microbiota affect the growth of transplanted endometrium (Chadchan et al., 2019; Yuan et al., 2018). It is unclear whether the mechanism involves the peritoneal microbiome, or associated growth factors. It explains observations in the human as the association of the endometriosis risk with diet as red meat (Yamamoto et al., 2018) or lipid intake or exercise since both diet (de Clercq et al., 2016) and exercise (Clark and Mach, 2016) affect gut microbiota.

### Sperm transport of infectious agents and pelvic and upper genital microbiota

Spermatozoa can transport some bacteria on their tails (Confino et al., 1987). It is surprising that the transport capacity by spermatozoa remains unexplored for most bacteria and viruses and intra- cellular replicating pro-karyotic organisms. It is therefore also unknown whether sperm transport plays a role in tubal damage by chlamydia, or in the pathogenesis of endometriosis.

Upper genital tract and peritoneal cavity microbiome by sperm transport of bacteria obviously are at best only one factor in the pathophysiology of endometriosis since the disease exists in the absence of sexual contact.

### Strength and weakness of these studies

The weakness of the association of endometriosis with upper genital tract and pelvic infections is that the articles are either microbiota studies, or population-based association studies, or studies investigating a specific infection. In most studies the diagnosis and type of endometriosis is poorly described. Except for studies describing severe deep or cystic ovarian endometriosis, it is rarely clear how subtle endometriosis were classified. Recognition of subtle lesions varies with the expertise and interest of the surgeon and it remains debated whether subtle endometriosis is pathology or whether it a physiological phenomenon occurring intermittently in all women (Koninckx et al., 2019). However, this does not invalidate the conclusion. On the contrary a stricter definition of endometriosis is expected to strengthen the conclusions of an association between endometriotic disease and infection.

### A publication bias is likely since studies with a negative outcome risk are less likely to be published.

Studies on PID and of the natural defence mechanisms are hampered by the inaccurate clinical diagnosis of PID, except for gonorrhoea with severe symptoms. An accurate diagnosis of PID requires a diagnostic laparoscopy and the identification of the infectious agents, which requires advanced laboratory techniques for some viruses and some bacteria. In addition, PID is often a poly-viral and/ or poly-bacterial infection.

## Conclusions

**Figure 1:**
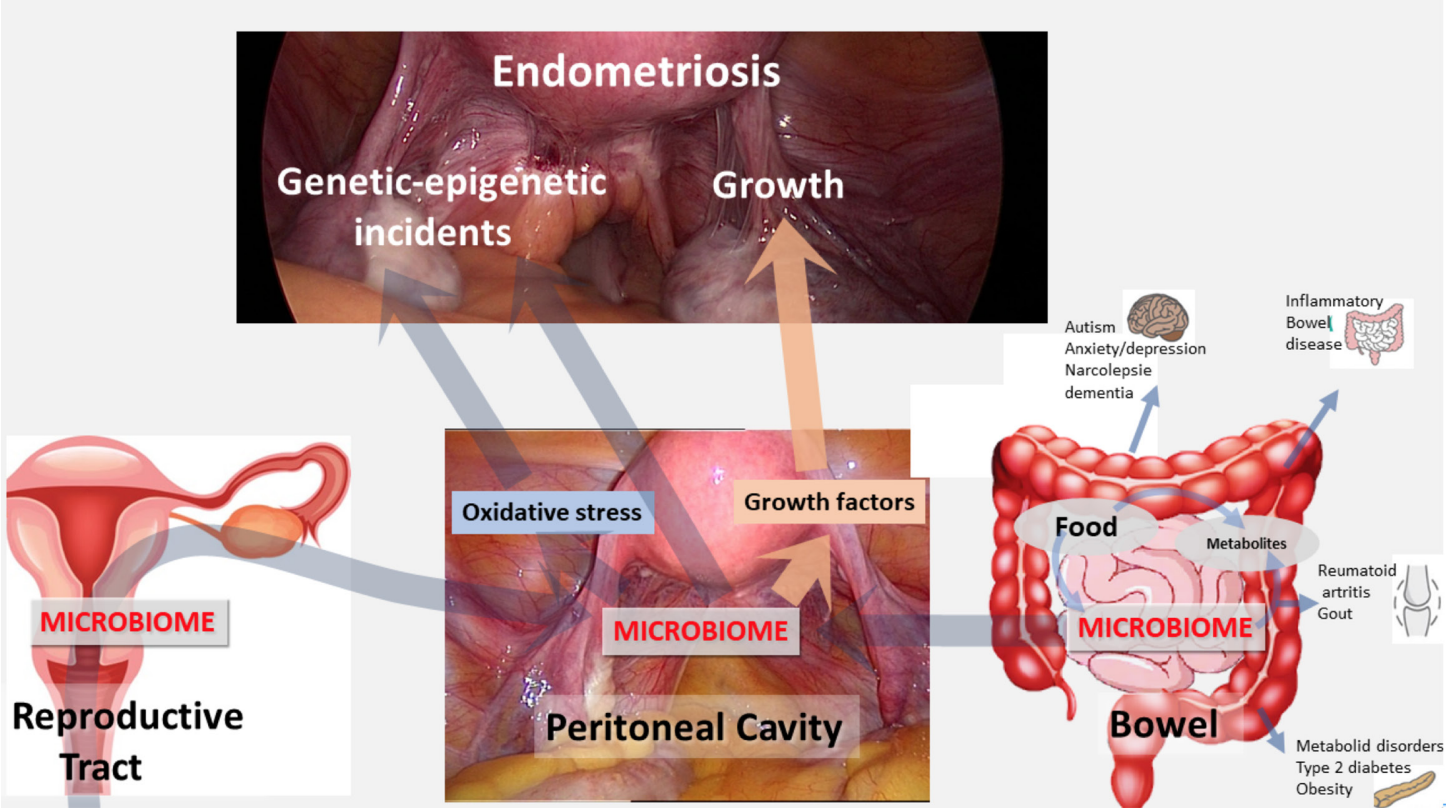
The peritoneal microbiome results from the uterine and upper-genital tract microbiome and the gut microbiome. The peritoneal microbiome can cause endometriosis by inducing genetic epigenetic incidents either directly or by increasing the oxidative stress. The peritoneal microbiome also can increase endometriosis growth through growth factors and immunologic changes. This explains why the gut microbiome, which is influenced by food intake and exercise, can influence the induction and growth of endometriosis, besides many other effects.

In conclusion, the uterine, tubal and pelvic cavities are not sterile, but they are environments with a specific immunity and microbiome. This is not surprising considering the connection to the outside world and the potential transport of micro- organisms by spermatozoa. The contribution of transmural migration of the gut microbiota is unclear. Endometriosis is associated with alterations in the microbiome of the upper-genital tract and the peritoneal cavity, with an increase in mollicutes and with HPV virus in the lesions. These infections have the potential to initiate endometriosis by causing genetic-epigenetic incidents and to contribute to the growth of endometriosis (Figure 1). Understanding the mechanism of upper genital tract infection and microbiota and the interplay with the gut microbiota is expected to lead to new therapies for prevention of endometriosis growth and recurrences.
